# Factors Associated with Long-Term Sickness Absence Due to Mental Disorders: A Cohort Study of 7.112 Patients during the Spanish Economic Crisis

**DOI:** 10.1371/journal.pone.0146382

**Published:** 2016-01-05

**Authors:** Eva Real, Lluís Jover, Ricard Verdaguer, Antoni Griera, Cinto Segalàs, Pino Alonso, Fernando Contreras, Antoni Arteman, José M. Menchón

**Affiliations:** 1 Psychiatry Department, Bellvitge University Hospital, Bellvitge Biomedical Research Institute (IDIBELL), Barcelona, Spain; 2 Carlos III Health Institute, Centro de Investigación Biomédica en Red de Salud Mental (CIBERSAM), Barcelona, Spain; 3 Biostatistics Unit, Department of Public Health, Faculty of Medicine, University of Barcelona, Barcelona, Spain; 4 Egarsat, Mutual Society for Work Accidents and Work-Related Illnesses n° 276, Spain; 5 SSM–Serveis de Salut Mental S.L. Comprehensive Health Services for Sick Doctors, Galatea Foundation, Barcelona, Spain; College of Agricultural Sciences, UNITED STATES

## Abstract

**Background:**

Mental health problems are very common and often lead to prolonged sickness absence, having serious economic repercussions for most European countries. Periods of economic crisis are important social phenomena that are assumed to increase sickness absence due to mental disorders, although research on this topic remains scarce. The aim of this study was to gather data on long-term sickness absence (and relapse) due to mental disorders in Spain during a period of considerable socio-economic crisis.

**Methods:**

Relationships were analyzed (using chi-squared tests and multivariate modelling via binary logistic regression) between clinical, social/employment-related and demographic factors associated and long-term sickness absence (>60 consecutive days) due to mental disorders in a cohort of 7112 Spanish patients during the period 2008–2012.

**Results:**

Older age, severe mental disorders, being self-employed, having a non-permanent contract, and working in the real estate and construction sector were associated with an increased probability of long-term sickness absence (gender had a mediating role with respect to some of these variables). Relapses were associated with short-term sick leave (return to work due to ‘improvement’) and with working in the transport sector and public administration.

**Conclusions:**

Aside from medical factors, other social/employment-related and demographic factors have a significant influence on the duration of sickness absence due to mental disorders.

## Introduction

When a person is unable to perform his or her usual work due to illness or injury, a period of temporary or permanent incapacity follows. Mental health problems are very common and often lead to prolonged sickness absence, much longer than would be expected by regulatory bodies [1]. In Spain, as in other European countries [2], this has a considerable economic impact on the health and social security system. According to the Organization for Economic Cooperation and Development (OECD), countries in the European Union spent on average 1.9% of GDP on disability pension benefits in 2011 [3]. Furthermore, between 33% and 50% of new applications for permanent disability status were based on the presence of a mental disorder, with the figure reaching 70% among young adults [4]. Consequently, there is a growing interest in developing effective strategies for managing and dealing with sickness absence due to mental disorders. However, the whole area of work incapacity is a complex one in which numerous factors other than purely medical issues are involved. Research on this topic in Spain is in its infancy and is particularly required in relation to sickness absence due to mental disorders, since this is a common and enduring problem and one that constitutes the primary reason for prolonged sick leave in many European countries [2,5–8].

The duration of sickness absence due to mental disorders has been linked to age [9], gender [10], level of education [2], various socio-economic indicators [8], the kind of benefits received while on sick leave [11], unfavourable employment conditions [12] and certain adverse psychosocial factors [13,14]. Periods of socio-economic crisis are known to have a considerable impact on rates of sickness absence, especially cases resulting from common mental disorders [15]. Spain is currently facing a crisis that has had particularly dramatic effects in terms of job losses and reduced average per capita incomes. Recent figures from the *World Top Incomes Database* [16] show that the average annual income of Spanish citizens has fallen by 13.4% since the start of the economic crisis (in 2007), with the figure for 2012 being on a par with that recorded in the year 2000 (€16,700 in 2012 vs. €19,300 in 2007). In addition, the unemployment rate has tripled (from 2,174,200 people during the first quarter of 2008 to 6,202,700 during the first quarter of 2013, according to the National Statistics Office) [17,18]. This is relevant since research has shown that people who lose their job during a period of crisis may end up with poorer general health compared with those who lose their job under other circumstances [19].

The present study sought to analyse sickness absence due to mental disorders in a large sample of Spanish patients (n = 7112), taking into account clinical, social/employment-related and demographic variables during a period of socio-economic crisis. The main aim was to determine which factors are associated with long-term sickness absence (and recurrence) due to mental disorders. To this end we conducted an analysis of the factors associated with long-term sick leave (>60 consecutive days) and developed a model to interpret the overall association. The ultimate goal was to identify the set of factors associated with long-term sick leave and the characteristics of those sickness absences most likely to recur.

## Method

### Incapacity for Work in Spain: The Study Context

In Spain, a person who is unable to work on health grounds is entitled to benefits for a maximum period of 12 months, which may, under exceptional circumstances, be extended to 18 months (when recovery seems likely). Sickness absences are managed by the National Institute for Social Security (INSS, in Spanish), normally in collaboration with the network of mutual societies for work accidents and work-related illnesses [1]. These mutual healthcare providers are responsible for the continuous medical follow-up of the patient, the aim being to avoid an unnecessary or prolonged sickness absence.

The present study was conducted through collaboration between the Psychiatry Service of Bellvitge University Hospital (Barcelona) and EGARSAT, one of Spain’s mutual healthcare providers. In collaboration with the National Institute for Social Security, EGARSAT manages and provides the employees of its affiliated companies with the healthcare required as a result of work accidents and work-related illnesses (including the payment of benefits and interventions designed to prevent work accidents or work-related illnesses). It also manages the benefits owing in the case of sickness absence due to non-occupational diseases and non-work-related injuries, whether for employees of affiliated companies or for self-employed workers who have opted in to the EGARSAT insurance scheme. In these cases, EGARSAT would also be responsible for the medical assessments associated with the payment of these benefits.

### Ethical Statement

Patients records were anonymized and de-identified prior to analysis. The study protocol was approved by the Ethics Committee of Bellvitge University Hospital. All procedures contributing to this work comply with the ethical standards of the relevant national and institutional committees and with the Helsinki Declaration of 1975, as revised in 2008.

### Sample

This was a cohort study of workers registered with a mutual healthcare provider and who had begun a period of sickness absence due to a mental disorder between 1 January 2008 and 31 December 2012. The sample included all patients who, being resident in the province of Barcelona, had a registered sickness absence of this kind lasting for more than one day (n = 13,005). Patients could have more than one period (or event) of sickness absence during the study period. Recurrent events of sick leave separated by fewer than 180 days (between the last day of a previous event and the first day of the next) were considered as relapses. These linked events were defined as an episode. When two events occurred more than 180 days apart they were considered as new (separate) episodes. Any repeated registrations of sickness absence were excluded (n = 102). The analysis was based on initial events of first episodes registered during the defined study period. Hence, any cases of sick leave that began prior to 1 July 2008 (n = 1647) or after 31 June 2011 (n = 953) were excluded. In this way we avoided taking into account possible relapses of events that had begun prior to the start of the study period, and we also ensured that we knew the end date of all events. By applying this criterion we excluded these relapses (and events related with them) (n = 97), as well as any episodes that were not initial events (n = 1094). The final sample therefore comprised 7112 patients.

### Variables and Recoding

Several kinds of variables were assessed: socio-demographic (age, gender, town/city of residence), clinical (psychiatric diagnosis according to ICD-9), employment-related (type of contract, type of social security contributions, and sector in which employed, coded according to the National Classification of Economic Activities, CNAE 2009), and those related to the sickness absence (duration, reason for end of sick leave). Some of these variables had to be recoded in order to make the analysis easier to perform. The psychiatric diagnosis (227 different disorders) was grouped and recoded into seven different subgroups. The various sectors of employment (457 in total) were regrouped into 13 subgroups through a slight modification of the classification used in the CNAE 2009. The recoding of these variables is shown in Table 1.

**Table 1:** Descriptive and comparative data for clinical and social/employment-related variables in a sample of 7112 patients according to whether or not they presented long-term sickness absence.

		SA > 60 days							
	%	No		Yes		Chi-sq.	p-value	OR	95% CI
** Gender**									
Male	42.8	1751	(57.5)	1294	(42.5)	0.48	0.4887	1.020	(0.965–1.077)
Female	57.2	2372	(58.3)	1695	(41.7)				
**Age**									
≤30	19.7	929	(66.2)	475	(33.8)	134.6	<0.001		
30–40	33.2	1424	(60.4)	935	(39.6)				
40–50	26.1	1085	(58.5)	769	(41.5)				
>50	21.0	684	(45.8)	808	(54.2)				
** Year**									
2008	18.0	734	(57.3)	547	(42.7)	48.81	<0.0001		
2009	36.5	1436	(55.4)	1157	(44.6)				
2010	29.3	1179	(56.5)	906	(43.5)				
2011	16.2	774	(67.1)	379	(32.9)				
** Quarter in which SA began**									
Jan-March	27.6	1143	(58.2)	821	(41.8)	9.38	0.025		
April-June	24.3	1051	(60.8)	677	(39.2)				
July-September	22.4	903	(56.7)	689	(43.3)				
Oct-December	25.7	1026	(56.1)	802	(43.9)				
**Half of month in which SA began**									
First	50.9	2098	(58)	1522	(42)	0.001	0.9769	1.001	(0.948–1.057)
Second	49.1	2025	(58)	1467	(42)				
** Type of social security contributions**									
General scheme	85.0	3857	(63.8)	2187	(36.2)	563.9	<0.0001	0.482	(0.459–0.506)
Self-employed	15.0	266	(24.9)	802	(75.1)				
**Type of contract: duration**^**1**^									
Permanent	77.5	2934	(65.2)	1566	(34.8)	14.91	0.0001	0.857	(0.793–0.925)
Fixed-term	22.5	776	(59.4)	531	(40.6)				
** Type of contract: hours**^**1**^									
Full-time	76.6	2877	(64.6)	1574	(35.4)	8.68	0.013		
Part-time	15.9	583	(63.2)	339	(36.8)				
Work and services	7.5	250	(57.6)	184	(42.4)				
**Psychiatric diagnosis**^**2**^									
<Psychotic dis.	1.2	31	(36)	55	(64)	332.4	<0.0001		
Affective dis.	15.8	417	(37.1)	706	(62.9)				
Anxiety dis.	69.8	3110	(62.7)	1853	(37.3)				
Adaptive dis.	6.6	293	(62.7)	174	(37.3)				
Substance dependence	1.2	32	(38.1)	52	(61.9)				
Personality dis.	1.1	20	(26)	57	(74)				
Other	4.4	220	(70.5)	92	(29.5)				
**Economic activity of employer**^**3**^									
Manufacturing industry	24.0	1124	(66)	579	(34)	151.0	<0.0001		
Energy and water supply	1.4	67	(67.7)	32	(32.3)				
Construction, real estate	10.3	326	(44.4)	408	(55.6)				
Wholesale or retail trade	20.5	814	(55.9)	641	(44.1)				
Transport and warehousing	4.9	188	(54.5)	157	(45.5)				
Hotel and catering	8.0	299	(52.4)	272	(47.6)				
Administrative and auxiliary services auxiliaries	10.9	418	(53.8)	359	(46.2)				
Financial and insurance	0.7	22	(42.3)	30	(57.7)				
Scientific, technical and education	5.0	222	(62)	136	(38)				
Public administration	5.4	266	(69.6)	116	(30.4)				
Health and social services	4.7	200	(60.2)	132	(39.8)				
Other (domestic staff)	4.2	172	(57.7)	126	(42.3)				
**Relapse**									
No	91.6	3707	(56.9)	2806	(43.1)	35.3	<0.0001	1.410	(1.246–1.596)
Yes	8.4	416	(69.4)	183	(30.6)				
**Reason for end of SA**									
Administrative	5.8	96	(23.2)	318	(76.8)	441.8	<0.0001		
Improvement	92.0	4024	(61.5)	2517	(38.5)				
Permanent disability	2.2	3	(1.9)	154	(98.1)				

SA: sickness absence

Values of SA = number of patients (percentage of patients within the group)

^1^Data correspond only to employees covered by the general social security scheme (85% of cases, n = 5807)

^2^According to the International Classification of Diseases (ICD-9)

^3^According to the National Classification of Economic Activities (CNAE)

OR, odds ratio

CI, confidence interval

The period of the year was assessed because it has been observed by mutual healthcare providers some kind of seasonal nature in the rates of sickness leave (i.e: higher rates in January and lower in July-August). This apparent trend has not been analyzed before and the role of several factors as holiday periods in Spain cannot be discarded (these periods may vary the administrative management processes, the timings in workload or more complex issues). Moreover, potential changes in the season of interview have been considered as a limitation in research similar to ours [20]. The half of the month was assessed because, taking into account that most contracts start the first day of the month, one might expect that sick leave began more frequently within the second half of the month in vulnerable people (who would need to stop working some days after joining a new job), although it is speculative. This variable has been used in previous reports on the association of anxiety disorders on sickness absence in Spain, too [2].

Socio-economic and demographic information was also gathered regarding the town/city of residence for all the patients included in the study (gross household income, rate of unemployment, population density, proportion of non-EU immigrants, proportion of inhabitants with secondary or higher education, among others). This information was obtained through the Catalan Office for Statistics (IDESCAT), which is the most reliable source of population data for the geographical area covered by the study. Finally, the dependent variable (the duration of sickness absence) was dichotomized as either long-term or short-term using 60 days as the dividing point. Several clinical and administrative reasons support this cut-off point in our setting. The most relevant one is that, according to the INSS, most mental disorders should be recovered within that period of time [21], with management and financial implications. The 60 days cut-off has been also used in previous reports [22].

### Analysis

The first step involved encrypting (anonymizing) all codes that served to identify patients. This was followed by a pre-analysis and an examination of data quality (identification and correction of anomalous values and/or outliers). We then conducted a full descriptive analysis of all the variables that might be of interest in relation to sickness absence, this being based on the total sample (n = 7112). The dependent variable was the duration of sickness absence, which we dichotomized as either long-term or short-term. The chi-square test of independence was used to assess the possible association between the variables of interest and the duration of sickness absence.

Multivariate binary logistic regression was then used to modelize the probability of long-term sickness absence. The model was built using hierarchical backward selection [23], applying the likelihood ratio test to assess, first, the presence of significant interactions. Having identified the interactions that should remain in the model and the variables implied in them, the criterion of no change in the model’s parameter estimates was used to eliminate other covariables. For this procedure we began with a saturated model that included all the variables considered to be potentially relevant: gender, age group, psychiatric diagnosis, economic activity of the employer, type of social security contributions, and period of the year (quarter) and month (first or second half) in which the sickness absence began, as well as the first-order interactions between these variables. For the final selected model we estimated the corresponding confidence intervals for each variable, or for their combinations in the event of an interaction; these calculations are presented in the form of odds ratios (OR) in order to facilitate their interpretation. The model selection was performed using a random sample of 70% of the observations, with the goodness of fit being tested to ensure that it was maintained when the remaining observations were included. The final parameter estimates of the model were calculated using all the available observations. The aim was 1) to estimate the associations between the variables of interest and the risk of sickness absence being long-term, adjusted for the remaining variables included in the model, and 2) to evaluate whether any of these associations were modulated by other factors (interaction terms). All statistical analyses were performed using IBM SPSS Statistics v.22.

## Results

Of the 7112 persons with a recorded sickness absence due to a mental disorder during the period 2008–2011, 2989 (42%) presented a long-term absence and 599 (8.4%) relapsed during the same period. The majority of sickness absences were due to anxiety disorders (4963, 69.8%) and ended as a result of clinical improvement (6541, 92%); in most cases no specialist psychiatric assessment was required (6091, 85.6%). The distribution of the sample with respect to the other variables studied (gender, age, psychiatric diagnosis, durational and employment-related factors associated with the sickness absence, and socio-economic indicators for the patients’ town/city of residence) is shown in Table 1.

The comparative analysis of short- and long-term sickness absences revealed differences in terms of age but not by gender. Sick leave was progressively more likely to be long-term as patients got older, with the probability being lowest in the under 30 age group and highest among those aged 50 (33.8% vs. 54.2%; χ^2^ = 134.6, df = 3, p < 0.001). The type of social security contributions showed a robust association with the duration of sick leave, with self-employed workers being twice as likely as those under the general insurance scheme to present a long-term sickness absence (OR = 2.07, CI 1.98–2.18). A predominance of short-term sickness absences was observed in the quarter prior to the summer holiday period (χ^2^ = 9.38, df = 3, p < 0.025), as well as in final year of the study period (χ^2^ = 48.81, df = 3, p < 0.001). Other employment-related factors were also associated with the duration of sick leave. Specifically, among patients covered by the general social security scheme, long-term sickness absence was significantly associated with having a fixed-term contract (χ^2^ = 14.9, df = 1, p < 0.001) or a contract for ‘work and services’ (χ^2^ = 8.7, df = 2, p = 0.013).

Three out of every four long-term sickness absences ended for administrative reasons, whereas the majority of short-term absences ended due to clinical improvement (χ^2^ = 1524.5, df = 7, p < 0.001). Patients who presented a relapse during the study period were more likely to have originally been assigned to the group of short-term sickness absence (χ^2^ = 35.36, df = 1, p < 0.001). With respect to psychiatric diagnosis, affective disorders and personality disorders were more commonly associated with long-term sickness absences (followed by psychotic disorders and substance dependence), whereas anxiety disorders were more likely in the case of short-term sick leave (χ^2^ = 332.4, df = 6, p < 0.001). As regards the sector of employment, long-term sickness absences were strongly associated with the construction and real estate sector, whereas short-term sick leave was significantly more likely in the context of the manufacturing industry (χ^2^ = 151.0, df = 11, p < 0.001). The two sickness absence subgroups (short- vs. long-term) did not differ meaningfully in terms of the socio-economic characteristics of patients’ town/city of residence (see Table 2), since none of the statistically significant differences observed (e.g. household income) were of relevant magnitude.

**Table 2:** Comparison of socio-economic indicators for the town/city of residence in a sample of 7112 patients according to whether or not they presented long-term sickness absence.

	SA-ST				SA-LT			
	Mean	(± SE)	Median	IQR	Mean	(± SE)	Median	IQR
**Index of gross household per capita income**	97	(±0.19)	96	9	99	(±0.25)	96	8
**Population density (inhab./km**^**2**^**)**	5944	(±88.7)	3208	3471	5655	(±98.8)	3065	3240
**Percentage unemployed**	14.0%	(±0.04%)	14.8%	3.8%	14.0%	(±0.05%)	15.0%	4.1%
**Percentage non-EU immigrants**	10.2%	(±0.06%)	10.4%	4.7%	10.0%	(±0.07%)	10.0%	4.2%
**Percentage of inhabitants with secondary or higher education**	74.6%	(±0.06%)	73.6%	4.1%	74.9%	(±0.08%)	73.6%	4.6%

SA-ST: short-term sickness absence

SA-LT: long-term sickness absence

EU: European Union

SE: standard error

IQR: interquartile range

Relapse was inversely related to the duration of sick leave. Specifically, the probability of relapse was almost double among patients who originally presented a short-term as opposed to a long-term absence (OR_ST/LT_ = 1.72; CI: 1.44–2.06). Relapse was also associated with those sickness absences that ended due to ‘improvement’ (χ^2^ = 25.4, df = 2, p < 0.001), as well as with certain sectors of employment, namely public administration and transport (χ^2^ = 22.3, df = 11, p = 0.022). The results for the remaining comparisons regarding relapse are shown in Table 3.

**Table 3:** Descriptive and comparative data for clinical and social/employment-related variables in a sample of 7112 patients according to whether or not they presented a relapse.

		Relapse							
	%	No		Yes		Chi-sq.	p-value	OR	95% CI
** Gender**									
Male	42.8	2767	(90.9)	278	(9.1)	3.454	0.063	1.172	(0.991–1.366)
Female	57.2	3746	(92.11)	321	(7.89)				
**Age**									
≤30	19.7	1293	(92.1)	111	(7.9)	3.060	0.383		
30–40	33.2	2145	(90.9)	214	(9.1)				
40–50	26.1	1694	(91.4)	160	(8.6)				
>50	21.0	1378	(92.4)	114	(7.6)				
** Type of social security contributions**									
General scheme	85.0	5524	(91.4)	520	(8.6)	1.7	0.191	1.178	(0.922–1.508)
Self-employed	15.0	989	(92.6)	79	(7.4)				
**Reason for end of SA**									
Administrative	5.8	398	(96.1)	16	(3.9)	25.4	<0.001		
Improvement	92.0	5959	(91.1)	852	(8.9)				
Permanent disability	2.2	156	(99.4)	1	(0.6)				
**Psychiatric diagnosis**^**1**^									
Psychotic dis.	1.2	76	(88.4)	10	(11.6)	4.73	0.579		
Affective dis.	15.8	1023	(91.1)	100	(8.9)				
Anxiety dis.	69.8	4542	(91.5)	421	(8.5)				
Adaptive dis.	6.6	434	(92.9)	33	(7.1)				
Substance dependence	1.2	75	(89.3)	9	(10.7)				
Personality dis.	1.1	72	(93.5)	5	(6.5)				
Other	4.4	291	(93.3)	21	(6.7)				
**Economic activity of employer**^**2**^									
Manufacturing industry	24.0	1551	(91.1)	152	(8.9)	22.27	0.022		
Energy and water supply	1.4	90	(90.9)	9	(9.1)				
Wholesale and retail trade	20.5	1334	(91.7)	121	(8.3)				
Construction, real estate	10.3	683	(93.1)	51	(6.9)				
Transport and warehousing	4.9	303	(87.8)	42	(12.2)				
Hotel and catering	8.0	536	(93.9)	35	(6.1)				
Administrative and auxiliary services	10.9	715	(92)	62	(8)				
Financial and insurance	0.7	46	(88.5)	6	(11.5)				
Scientific, technical and education	5.0	329	(91.9)	29	(8.1)				
Public administration	5.4	337	(88.2)	45	(11.8)				
Health and social services	4.7	304	(91.6)	28	(8.4)				
Other (domestic staff)	4.2	281	(94.3)	17	(5.7)				
**Duration of SA**									
2–15 days	28.8	1814	(88.7)	232	(11.3)	43.58	<0.0001		
16–60 days	29.2	1893	(91.1)	184	(8.9)				
61 or more days	42.0	2806	(93.9)	183	(6.1)				
**Long-term SA**									
No	58.0	3707	(89.9)	416	(10.1)	35.36	<0.0001	1.721	(1.437–2.062)
Yes	42.0	2806	(93.9)	183	(6.1)				

SA: sickness absence

Values for relapse = number of patients (percentage of patents within group)

^1^According to the International Classification of Diseases (ICD-9)

^2^According to the National Classification of Economic Activities (CNAE)

OR: odds ratio

CI: confidence interval

Table 4 shows the results for the modelling of risk factors associated with long-term sickness absence. The final model considered the following main effects: type of social security contributions, age, gender, psychiatric diagnosis, economic activity of the employer, and half of the month (first vs. second) in which sick leave began (gender showed an interaction effect with both half of the month and the type of social security contributions). According to the model, working in the construction or real estate sector, older age, and being diagnosed with a personality disorder, a psychotic disorder or an affective disorder (in this order) increased the likelihood of a patient presenting a long-term sickness absence. The factor most strongly associated with long-term sick leave was the type of social security contributions, but its effect interacted with gender. When the three variables involved in the observed interaction effect (gender, type of social security contributions and half of the month in which sick leave began) were considered together, the highest risk of a long-term sickness absence corresponded to self-employed women who began their sick leave in the second half of the month. The influence of gender was much weaker among workers covered by the general social security scheme, and the effect of both the type of social security contributions and the half of the month in which sick leave began was less pronounced among men than women.

**Table 4:** Modelling of risk factors for long-term sickness absence due to mental disorders in a sample of 7112 patients assessed during the period 2008–2011.

Model Estimates					
Model parameter	Value	Standard error	Wald test p-value	OR	95% CI
Constant	1.301	0.1601	<0.001		
**Economic activity of employer**					
Manufacturing industry	-0.311	0.0789	0.000	0.73	0.63–0.86
Energy and water supply	-0.311	0.2289	0.175	0.73	0.47–1.15
Construction, real estate	0.323	0.1007	0.001	1.38	1.13–1.68
Transport and warehousing	-0.031	0.1309	0.810	0.97	0.75–1.25
Hotel and catering	0.149	0.1063	0.161	1.16	0.94–1.43
Administrative and auxiliary services	0.130	0.0950	0.172	1.14	0.95–1.37
Financial and insurance	-0.045	0.3127	0.886	0.96	0.52–1.77
Scientific, technical and education	-0.141	0.1279	0.269	0.87	0.68–1.12
Public administration	-0.374	0.1282	0.004	0.69	0.54–0.88
Health and social services	-0.003	0.1303	0.980	1.00	0.77–1.29
Other (other and domestic staff)	-0.098	0.1390	0.479	0.91	0.69–1.19
Wholesale and retail trade	reference				
**ICD-9**					
Psychotic disorders	1.082	0.2518	<0.001	2.95	1.80–4,83
Affective disorders	0.847	0.1193	<0.001	2.33	1.85–2.95
Anxiety disorders	0.007	0.1044	0.947	1.01	0.82–1.24
Substance dependence	0.766	0.2571	0.003	2.15	1.30–3,56
Personality disorders	1.348	0.2890	<0.001	3,85	2.19–6,78
Other	-0.322	0.1634	0.049	0.72	0.53–1.00
Adaptive disorders	reference				
**Age Group**					
≤30	-0.608	0.0829	<0.001	0.54	0.46–0.64
30–40	-0.395	0.0721	<0.001	0.67	0.59–0.78
40–50	-0.402	0.0756	<0.001	0.67	0.58–0.78
>50	reference				
**Type of social security contributions**					
General scheme	-1.522	0.1134	<0.001		
Self-employed	reference				
**Gender**					
Male	-0.448	0.1565	0.004		
Female	reference				
**Half of month in which SA began**					
First	-0.095	0.0681	0.164		
Second	reference				
**Gender x Type of social security contributions**					
Male-General scheme	0.292	0.1564	0.062		
**Gender x Half of month SA began**					
Male-First half	0.238	0.1040	0.022		

Dependent variable: long-term sickness absence (>60 days)

Model (intersection): Type of social security contributions, gender, age, psychiatric diagnosis, economic activity of employer, half of month in which the sickness absence began, type of contributions*gender, gender*half of month

OR: odds ratio; CI: confidence interval

The model for the likelihood of relapse included the economic activity of the employer, the age of the patient (4 categories) and the type of social security contributions (the latter two variables showed an interaction effect). With respect to the economic activity of the employer and taking the wholesale/retail trade as a reference, people working in public administration and the transport sector were more likely to present a relapse. As regards age and the type of social security contributions (and according to the estimated model), patients in the middle age bracket (30–50 years) were more likely to relapse if they were covered by the general scheme rather than being self-employed. However, this trend disappeared among older workers, among whom no difference was observed, whereas the opposite trend was found in the youngest age group, that is, relapse was significantly more likely among self-employed workers (data not shown).

## Discussion

To our knowledge this is the largest study to date of sickness absence due to mental disorders in Spain. The results show that older age, severe mental health problems (such as psychotic or affective disorders), being self-employed, having a non-permanent contract, and working in the real estate and construction sector were associated with an increased likelihood of long-term sickness absence. In addition, the effect on the duration of sickness absence shown by two variables, namely the type of social security contributions and the half of the month in which sick leave began, was mediated by gender, such that self-employed women who began their sick leave in the second half of the month were more likely to present a long-term sickness absence. Our analysis also showed that relapses were more closely associated with short-term sick leave, with sickness absences that ended due to ‘improvement’ in the patient, and with workers in the transport and public administration sectors.

The main strengths of the study are as follows: 1) the sample size is considerable; 2) the psychiatric diagnoses had been certified by a physician (either a general practitioner or psychiatrist), thus avoiding the bias that might derive from the use of self-reports [21]; 3) the diagnoses were based on a standard and internationally recognized classification system (ICD-9); and 4) the socio-economic and demographic data obtained retrospectively are objective and come from a recognized public register (IDESCAT). There are also a number of limitations. First, the cross-sectional nature of the study prevents us from establishing causal relationships between the variables of interest. Second, the results are only representative of patients seen within the network of mutual societies for work accidents and work-related illnesses and cannot be generalized to sickness absence in the Spanish population as a whole. It should be noted, however, that over 75% of the working population in Spain is covered by one of these mutual health providers [24]. A final limitation is that the sample was restricted to the province of Barcelona in order to achieve the degree of homogeneity required for an adequate interpretation of the data, although this may also, in part, limit the generalizability of the findings.

The most common cause of sickness absence in our sample was anxiety disorders (70% of cases). This finding is consistent with other research carried out in our country [25], which similarly found that this kind of mental health problem was already a much more frequent cause of sick leave than were other mental disorders, before the beginning of the economic crisis. Very recent evidence indicates that there is a connection between economic decline and mental distress in Spain. It has been reported an increase in the prevalence of poor mental health among men between 2006–2007 and 2011–2012 [26] as well as in primary care attendance for most types of mental health disorders (including anxiety disorders) [20]. Moreover, a raise in suicidal behavior has been observed, mainly in the Mediterranean and Northern areas of Spain, amongst males and amongst those of working age [27]. Navarro-Mateu recently reported that twelve-month prevalence in Murcia (one of the 17 Spain’s Autonomous Communities) during the period of the economic crisis was significantly superior of that described ten years before for any disorder and for any anxiety disorder (9.7; 7.6 to 12.2 vs 5.9; 4.5 to 7.3) [28]. Although differences between Spanish Autonomous Communities in mental health issues have been described [29], the results of this study may be considered an indirect measure of the impact of the economic situation in Spanish citizens’ mental health, and in the need for sickness absence as a consequence. In the present sample, anxiety disorders generally produced only a short-term sickness absence (only a third required long-term sick leave), whereas more severe mental health problems (psychotic or affective disorders) led, in two-thirds of cases, to a long-term absence. From a population point of view, however, it is easy to imagine that anxiety disorders might have a more negative impact in terms of health expenditure, due to their high incidence and tendency to recur [30,31].

A further noteworthy finding in this regard is that relapses were associated with short-term sickness absences that had ended due to ‘improvement’ in the patient, since these kinds of psychiatric disorders are generally considered to be milder. Relapses, however, imply a new period of sick leave and a prolongation of the incapacity for work. All this highlights the complexity of the study phenomenon and the need to examine in greater depth the various aspects of sickness absence.

Some previous studies have reported gender differences in relation to sickness absence due to mental disorders, namely that women were more likely to present such an absence and that men’s sick leave lasted longer [10,32]. However, more recent studies have found no gender differences in this regard [8]. In our sample, gender did not have a significant independent influence on the duration of sick leave. However, it did mediate the effect that some other variables (such as the type of social security contributions and the half of the month in which sick leave began) had on the duration of sickness absence. Specifically, we found that self-employed women were much more likely to present a long-term sickness absence. Self-employed workers in Spain are in a more vulnerable situation than are employees covered by general social security contributions, since the former are not entitled to unemployment benefit and the benefits they receive in the event of illness or retirement are also less generous in economic terms. Our results confirm the observation made by many professionals regarding the association between prolonged sickness absence and women working in unskilled, low pay jobs. The interaction effect observed here might explain the discrepancies found in the literature regarding the role of gender, although we cannot rule out the possibility that this influence may differ according to the area of work.

Age had a direct effect on the duration of sickness absence. Patients over the age of 50 were the highest risk group for presenting a long-term sickness absence and for receiving a permanent disability pension (data not shown). These results are consistent with those of the majority of studies, which indicate that both the duration of sickness absence and the likelihood of a person being granted permanent disability status increase with older age [7,9]. In our sample only 2.8% of patients ended up receiving a permanent disability pension, a figure that contrasts sharply with those reported in Scandinavian countries: 16.6% in the study by Gjesdal *et al*. (2003) and even higher in subsequent research [33]. These variations could be due to differences in disability benefit systems between countries. For instance, it is possible that Scandinavian countries, which have a more established tradition and culture surrounding occupational health, pay greater attention to assessing the demands of the labour market and may recognize small reductions in the capacity to work as meriting some form of compensatory benefits [33]. Whatever the case, the results should be interpreted with caution when making international comparisons.

The type of employment contract was also associated with the duration of sickness absence. In our sample, long-term sick leave was more likely when the person had a fixed-term contract or a contract for ‘work and services’. This is consistent with previous studies reporting that workers without a permanent contract constitute a vulnerable group within the working population [12]. One of the greatest difficulties in studying incapacity for work is that many factors which are not strictly medical in nature have an influence on its development and maintenance [14]. Indeed, psychosocial factors have been shown to be predictors of long-term sickness absence due to both mental disorders and physical illness [34]. In times of economic difficulty, mental health problems tend to increase, but workers also exercise greater caution when requesting sick leave. An observational study carried out in The Netherlands demonstrated that the incidence of sickness absence (both in general and due to mental health) decreased slightly in all sectors between 2004 and 2009, coinciding with the economic recession in that country. However, the rate of sickness absence then increased again in 2010 as the economy began to pick up [15]. A similar pattern can be observed in our data, which cover the first four years of the economic crisis in Spain. The job losses and financial problems resulting from the crisis have been accompanied by a highly significant increase in the incidence of mental health problems in the Spanish population during this period [35]. However, the incidence of long-term sickness absence decreased significantly in our sample for the years 2010 and 2011 (compared with 2008 and 2009). This finding could be due to several factors: to a reluctance on the part of workers to request sick leave due to a fear of losing their job, to improved therapeutic approaches to mental disorders, leading to the early resolution of clinical problems, or to more detailed and rigorous monitoring of sickness absences by the responsible bodies (i.e. the National Institute for Social Security and the mutual societies for work accidents and work-related illnesses). From a social and employment perspective, it is striking that the sectors with the highest rates of long-term sickness absence in our sample were construction and real estate (the rate being much higher than would be expected from a merely statistical point of view). One can readily imagine that people working in the sectors most affected by the economic crisis would present the highest rates of long-term sickness absence. Various factors may be contributing to this association, since the sustained presence of environmental stressors usually serves to maintain psychological problems, thereby leading to longer periods of sick leave. This conclusion is supported by the results of a meta-analysis of longitudinal studies published between 1994 and 2005 which found important associations between tension in the workplace and mental disorders [36]. Research in Spain is very scarce on this specific topic and does not enable reliable conclusions to be drawn.

Although the variables included in our predictive model showed robust associations with the duration of sickness absence, the predictive capacity was very modest. There could be several reasons for this. On the one hand, the predictive capacity of a model seems to depend on the setting, such that our model may have lost power by including all sectors of employment. Alternatively, personality characteristics (and other psychosocial factors that were not considered) may be influencing the duration of sickness absence [33]. Very few studies have sought to build predictive models for long-term sickness absence. Roelen *et al*. developed a model that was able, in 73% of cases, to identify hospital workers at high (or low) risk of long-term sickness absence [37]. However, this model was not adequately validated when applied to a sample of nurses in another country [38].

Studying sickness absence is a complex task, but research is necessary because prolonged sick leave has been associated with greater difficulties returning to work, increased social isolation, poverty [39] and a heightened mortality risk [40]. Added to that is the controversy regarding the effectiveness of therapeutic interventions in the workplace designed to reduce the duration of sickness absence, since not all studies have found them to be clearly beneficial [41–43]. In fact, some studies have demonstrated that recipients of a return-to-work intervention (in comparison with those whose cases are managed conventionally) take longer to return to work and are more likely to be receiving sickness benefits at one-year follow-up [44]. This could be due to the dual role exercised by physicians in many countries (including Spain), in other words, they act both therapeutically and as case managers of sickness absences. There is an inherent paradox to this situation which could be interfering with the potential clinical benefits of therapeutic intervention (as well as with the doctor-patient relationship [41], thereby highlighting the need for the two roles to be fulfilled by different professionals (or official bodies). Aside from the differences between countries with respect to the system of sickness benefits, there is also debate regarding the cut-off point that should be used to define long-term sickness absence. Several cut-offs have been described in the literature: >7 accumulated days/year [45], >30 accumulated days/year [37], >15 consecutive days [33], >60 consecutive days [22], >90 consecutive days [8] and >196 consecutive days [7], among others. This lack of consensus could explain, at least in part, the differences observed in the literature to date, and highlights the need for a better understanding of the mechanisms that trigger and maintain sickness absence due to mental disorders.

## Conclusion

The aim of this study was to gather data on long-term sickness absence (and relapse) due to mental disorders in Spain during a period of considerable socio-economic crisis. The analysis shows that in addition to medical factors, other demographic and social/employment-related variables have a significant influence on the duration of sickness absence. There is a need for further studies that include the adequate tools and measures for analysing adverse factors associated with the workplace, not only because such factors may be of relevance but also because very little research of this kind has so far been conducted in Spain. Although this is a challenging task, a better understanding of sickness absence due to mental disorders could help to limit the negative repercussions of the situation, both for the patient and the national economy.
